# *N*-(Fluoren-9-ylmethoxy­carbon­yl)-l-isoleucine

**DOI:** 10.1107/S1600536808021855

**Published:** 2008-07-19

**Authors:** Kazuhiko Yamada, Daisuke Hashizume, Tadashi Shimizu, Kenzo Deguchi

**Affiliations:** a National Institute for Materials Science, 3-13 Sakura, Tsukuba 305-0003, Japan; b Advanced Technology Support Division, RIKEN, 2-1 Hirosawa, Wako, Saitama 351-0198, Japan

## Abstract

In the crystal structure of the title compound [systematic name fluoren-9-ylmethyl *N*-(1-carb­oxy-2-methyl­butyl)carbamate], C_21_H_23_NO_4_, the mol­ecular plane of the O=C—NH—C_α_ unit is slightly pyramidalized. The N atom deviates from the basal plane by 0.2086 (12) Å. The O=C—N—C_α_ torsion angle is −17.2 (2)°, and the C—N and O=C bond lengths are 1.3675 (17) and 1.2122 (17) Å, respectively. Apparently the character of the *sp* ^2^ hybrids of the mol­ecular plane is, to some extent, reduced. The crystal structure exhibits two inter­molecular hydrogen bonds (O—H⋯O and N—H⋯O), in which the hydr­oxy O atom acts as a donor to the carbonyl group and an acceptor of the amide group, respectively.

## Related literature

For related literature on the crystal structures of *N*-α-fluoren-9-ylmethoxy­carbonyl-protected amino acids, see: Valle *et al.* (1984 ▶); Yamada, Hashizume & Shimizu (2008 ▶); Yamada, Hashizume, Shimizu *et al.* (2008 ▶).
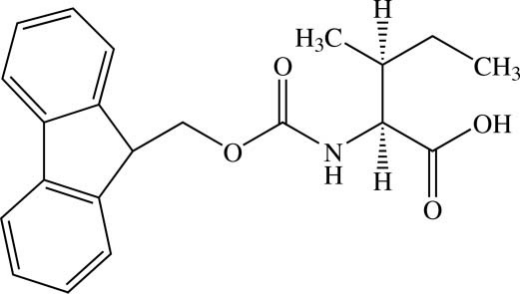


## Experimental

### 

#### Crystal data

C_21_H_23_NO_4_
*M* *_r_* = 353.40
Orthorhombic, 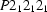

*a* = 5.3337 (2) Å
*b* = 13.6965 (4) Å
*c* = 25.2514 (9) Å
*V* = 1844.70 (11) Å^3^
*Z* = 4
Mo *K*α radiation
μ = 0.09 mm^−1^
*T* = 90 K
0.77 × 0.06 × 0.04 mm

#### Data collection

Rigaku AFC-8 diffractometer with Saturn70 CCD
Absorption correction: none
19825 measured reflections
3351 independent reflections
2993 reflections with *I* > 2σ(*I*)
*R*_int_ = 0.043

#### Refinement

*R*[*F* ^2^ > 2σ(*F* ^2^)] = 0.034
*wR*(*F* ^2^) = 0.084
*S* = 1.03
3351 reflections
327 parameters
All H-atom parameters refined
Δρ_max_ = 0.23 e Å^−3^
Δρ_min_ = −0.18 e Å^−3^

### 

Data collection: *CrystalClear* (Rigaku/MSC, 2005 ▶); cell refinement: *HKL-2000* (Otwinowski & Minor, 1997 ▶); data reduction: *HKL-2000*; program(s) used to solve structure: *SIR2004* (Burla *et al.*, 2005 ▶); program(s) used to refine structure: *SHELXL97* (Sheldrick, 2008 ▶); molecular graphics: *ORTEP-3 for Windows* (Farrugia, 1997 ▶); software used to prepare material for publication: *SHELXL97*.

## Supplementary Material

Crystal structure: contains datablocks I, global. DOI: 10.1107/S1600536808021855/is2313sup1.cif

Structure factors: contains datablocks I. DOI: 10.1107/S1600536808021855/is2313Isup2.hkl

Additional supplementary materials:  crystallographic information; 3D view; checkCIF report

## Figures and Tables

**Table 1:** Hydrogen-bond geometry (Å, °)

*D*—H⋯*A*	*D*—H	H⋯*A*	*D*⋯*A*	*D*—H⋯*A*
O1—H1H⋯O2^i^	0.88 (2)	1.77 (2)	2.6511 (14)	176 (2)
N1—H1N⋯O1^ii^	0.88 (2)	2.18 (2)	3.0433 (16)	167.6 (17)

Symmetry codes: (i) 
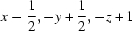
; (ii) 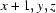.

**Fig. 1.**
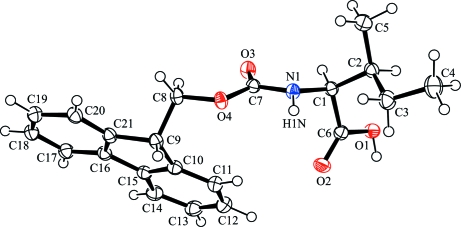
A view of the molecular structure of (I), showing the atom labeling scheme. Displacement ellipsoids are drawn at the 50% probability level.

**Fig. 2.**
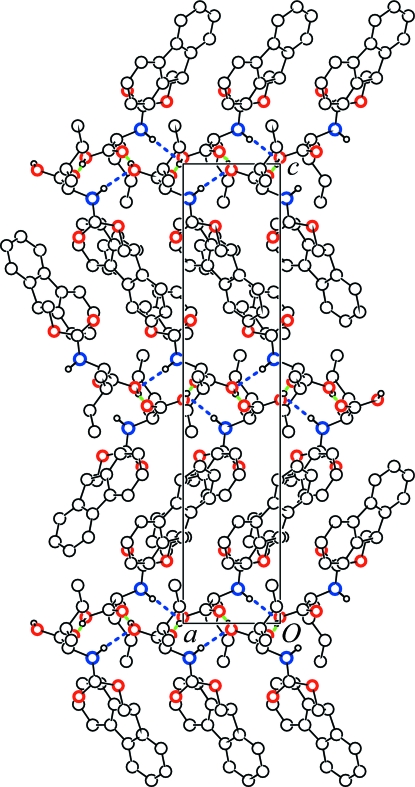
A packing diagram of (I). The hydrogen atoms were omitted for clarity, except for those forming the hydrogen bonds. The hydrogen bonds are shown as blue and green broken lines for N—H···O and O—H···O bonds, respectively.

## supplementary crystallographic information

### Comment

The fluoren-9-ylmethoxycarbonyl (Fmoc) group is commonly used for protecting the terminal amine of the peptide for the current solid-phase peptide synthesis protocol. This is because cleavage of the Fmoc protecting group is easily achieved by mild basis conditions, *e.g*., piperidine, but it is very stable under acidic conditions. The crystal structures of *N*-α-Fmoc-protected-L-alanine monohydrate (Valle *et al.*, 1984), -*O*-*t*-butyl-L-serine (Yamada, Hashizume, Shimizu, Ohiki & Yokoyama, 2008) and –L-leucine (Yamada, Hashizume & Shimizu, 2008) have been studied. In this communication, we will report the structure of *N*-α-Fmoc-L-isoleucine, (I) (Fig. 1).

It is interesting to compare the present structure with that of the analog of the title compound, *i.e*., *N*-α-Fmoc-protected-L-leucine, (II). A large fraction of the bond distances and angles, and torsion angles of (I) are consistent with those of (II) except for the following points. First, the orientation of the carboxyl group around the C1—C6 bond is found to be opposite. The torsion angle of O2—C6—C1—N1 for (I) is -6.3 (2)°, while the corresponding angle of (II) is 159.29 (17)°. Second, the angle between the fluorine ring and the NC(=O)O plane is quite different. For example, the torsion angle of C7—O4—C8—C9 and O4—C8—C9—C10 are 121.17 (13) and -73.17 (14)°, respectively, for (I). The corresponding torsion angles of (II), on the other hand, are 93.78 (16) and 60.54 (17)°, respectively. Third, it can be seen that the O3—C7—N1—C1 plane of (I) is slightly pyramided. The N1 atom deviates from the basal plane (C1, C7, H1N) by 0.2086 (12) Å. Moreover, the distances of the N1—C7 and O3—C7 bonds are 1.3675 (17) and 1.2122 (17) Å, respectively, which are approximately 0.026 longer and 0.010 Å shorter than the corresponding bond lengths of (II), respectively. Apparently, the *sp*^2^ character of the N1 atom is, to some extent, reduced.

In addition, hydrogen-bond environments are slightly different between the two Fmoc-protected L-amino acids. The crystal of (I) contains two intermolecular hydrogen bonds (Table 1), while that of (II) has three hydrogen bonds. For (I), atom O1 forms two hydrogen bonds with O2 and N1, as shown in Figure 2. The molecules, which related by translation along the *a* axis are assembled *via* the N1—H1N···O1 hydrogen bonds to form a one dimensional tape structure. The tapes around the 2_1_ axis, which is parallel to the *a* axis, are joined together, then the column structure is formed.

### Experimental

A powdered sample of the title compound (I) was purchased from Wako Pure Chemical Industries, Ltd. (Osaka, Japan) and was used for the sample preparation without further purifications. Colorless needle like crystals of (I) were obtained from a saturated dichloromethane solution.

### Refinement

All H atoms were found on a difference map and were refined applying isotropic temperature factors.

The refined C—H, N—H or O—H dimensions are in the normal range: 0.975 (19)–1.015 (19) Å and 104.8 (10)–113.8 (11)° for C—H and C/N—C—H, respectively, for methyne; 0.942 (17)–1.01 (2) Å, 106.6 (13)–111.4 (12)° and 107.1 (18)–108.6 (14)° for C—H, C/O—C—H and H—C—H for methylene; 0.98 (3)–1.03 (3) Å, 109.5 (11)–112.8 (13)° and 104 (2)–111 (2)° for C—H, C—C—H and H—C—H, respectively, for methyl; 0.96 (2)–1.005 (19) Å and 117.7 (11)–123.1 (11)° for C—H and C—C—H, respectively, for aromatic; 0.88 (2) Å and 115.3 (12)–116.5 (12)° for N—H and C—N—H, respectively, for amide; 0.88 (2) Å and 108.7 (15)° for O—H and C—O—H, respectively, for hydroxyl. The range of the *U*_iso_ values of the H atoms are 0.016 (4)–0.067 (8) Å^2^.

In the absence of significant anomalous scattering effects, Friedel pairs have been merged.

### Crystal data

**Table:**

C_21_H_23_NO_4_	*F*_000_ = 752
*M**_r_* = 353.40	*D*_x_ = 1.272 Mg m^−^^3^
Orthorhombic, *P*2_1_2_1_2_1_	Mo *K*α radiation λ = 0.71073 Å
Hall symbol: P 2ac 2ab	Cell parameters from 19896 reflections
*a* = 5.3337 (2) Å	θ = 1.6–31.0º
*b* = 13.6965 (4) Å	µ = 0.09 mm^−^^1^
*c* = 25.2514 (9) Å	*T* = 90 K
*V* = 1844.70 (11) Å^3^	Needle, colourless
*Z* = 4	0.77 × 0.06 × 0.04 mm

### Data collection

**Table:**

Rigaku AFC-8 with Saturn70 CCD diffractometer	3351 independent reflections
Radiation source: fine-focus rotating anode	2993 reflections with *I* > 2σ(*I*)
Monochromator: confocal	*R*_int_ = 0.043
Detector resolution: 28.5714 pixels mm^-1^	θ_max_ = 31.0º
*T* = 90 K	θ_min_ = 1.6º
ω scans	*h* = −7→7
Absorption correction: none	*k* = −13→19
19825 measured reflections	*l* = −36→36

### Refinement

**Table:**

Refinement on *F*^2^	Secondary atom site location: difference Fourier map
Least-squares matrix: full	Hydrogen site location: difference Fourier map
*R*[*F*^2^ > 2σ(*F*^2^)] = 0.034	All H-atom parameters refined
*wR*(*F*^2^) = 0.084	*w* = 1/[σ^2^(*F*_o_^2^) + (0.0427*P*)^2^ + 0.2178*P*] where *P* = (*F*_o_^2^ + 2*F*_c_^2^)/3
*S* = 1.03	(Δ/σ)_max_ < 0.001
3351 reflections	Δρ_max_ = 0.23 e Å^−^^3^
327 parameters	Δρ_min_ = −0.18 e Å^−^^3^
Primary atom site location: structure-invariant direct methods	Extinction correction: none

### Special details

**Table:**

Experimental. All Friedel pairs were merged, and all *f*''s of containing atoms were set to zero.
Geometry. All e.s.d.'s (except the e.s.d. in the dihedral angle between two l.s. planes) are estimated using the full covariance matrix. The cell e.s.d.'s are taken into account individually in the estimation of e.s.d.'s in distances, angles and torsion angles; correlations between e.s.d.'s in cell parameters are only used when they are defined by crystal symmetry. An approximate (isotropic) treatment of cell e.s.d.'s is used for estimating e.s.d.'s involving l.s. planes.
Refinement. Refinement of *F*^2^ against all reflections. The weighted *R*-factor *wR* and goodness of fit *S* are based on *F*^2^, conventional *R*-factors *R* are based on *F*, with *F* set to zero for negative *F*^2^. The threshold expression of *F*^2^ > 2*σ*(*F*^2^) is used only for calculating *R*-factors(gt) *etc*. and is not relevant to the choice of reflections for refinement. *R*-factors based on *F*^2^ are statistically about twice as large as those based on *F*, and *R*- factors based on all data will be even larger.

### Fractional atomic coordinates and isotropic or equivalent isotropic displacement parameters (Å^2^)

**Table:**

	*x*	*y*	*z*	*U*_iso_*/*U*_eq_	
O1	−0.0029 (2)	0.12876 (8)	0.48657 (4)	0.0236 (2)	
H1H	−0.038 (4)	0.1865 (16)	0.5004 (8)	0.040 (6)*	
O2	0.3719 (2)	0.19896 (8)	0.47355 (4)	0.0287 (2)	
O3	0.4252 (2)	0.06814 (8)	0.34059 (4)	0.0254 (2)	
O4	0.83023 (19)	0.09577 (8)	0.36430 (4)	0.0219 (2)	
N1	0.5644 (2)	0.03926 (9)	0.42480 (4)	0.0201 (2)	
H1N	0.683 (4)	0.0588 (14)	0.4465 (7)	0.028 (5)*	
C1	0.3145 (3)	0.03122 (10)	0.44681 (5)	0.0198 (3)	
H1	0.201 (4)	0.0187 (13)	0.4152 (7)	0.029 (5)*	
C2	0.2942 (3)	−0.05077 (10)	0.48868 (5)	0.0221 (3)	
H2	0.124 (4)	−0.0497 (14)	0.5026 (7)	0.025 (5)*	
C3	0.4615 (4)	−0.03098 (13)	0.53677 (6)	0.0322 (4)	
H3A	0.641 (5)	−0.0353 (16)	0.5239 (9)	0.045 (6)*	
H3B	0.433 (4)	0.0382 (16)	0.5491 (8)	0.043 (6)*	
C4	0.4141 (5)	−0.10079 (16)	0.58280 (7)	0.0429 (5)	
H4A	0.524 (5)	−0.0821 (19)	0.6146 (10)	0.063 (7)*	
H4B	0.449 (5)	−0.1707 (17)	0.5734 (9)	0.050 (6)*	
H4C	0.237 (6)	−0.1000 (19)	0.5925 (10)	0.067 (8)*	
C5	0.3466 (4)	−0.14964 (12)	0.46325 (7)	0.0321 (4)	
H5A	0.235 (4)	−0.1587 (15)	0.4308 (9)	0.043 (6)*	
H5B	0.302 (4)	−0.2030 (14)	0.4887 (8)	0.034 (5)*	
H5C	0.526 (5)	−0.1554 (16)	0.4525 (8)	0.040 (6)*	
C6	0.2335 (3)	0.12921 (10)	0.47018 (5)	0.0209 (3)	
C7	0.5913 (3)	0.06860 (10)	0.37335 (5)	0.0195 (3)	
C8	0.8909 (3)	0.12808 (11)	0.31097 (5)	0.0214 (3)	
H8A	1.018 (4)	0.0822 (13)	0.2966 (7)	0.023 (4)*	
H8B	0.746 (3)	0.1242 (12)	0.2898 (6)	0.016 (4)*	
C9	0.9917 (3)	0.23248 (10)	0.31308 (5)	0.0218 (3)	
H9	1.136 (4)	0.2340 (14)	0.3393 (7)	0.033 (5)*	
C10	0.7894 (3)	0.30772 (10)	0.32413 (5)	0.0227 (3)	
C11	0.6461 (3)	0.32177 (11)	0.36923 (6)	0.0273 (3)	
H11	0.678 (4)	0.2802 (14)	0.4013 (7)	0.032 (5)*	
C12	0.4624 (3)	0.39449 (12)	0.36893 (6)	0.0307 (3)	
H12	0.365 (4)	0.4056 (14)	0.4011 (8)	0.037 (5)*	
C13	0.4225 (3)	0.45181 (12)	0.32390 (7)	0.0307 (3)	
H13	0.289 (4)	0.5019 (16)	0.3241 (8)	0.038 (5)*	
C14	0.5681 (3)	0.43873 (11)	0.27881 (6)	0.0272 (3)	
H14	0.540 (4)	0.4779 (14)	0.2461 (8)	0.033 (5)*	
C15	0.7524 (3)	0.36682 (10)	0.27912 (5)	0.0222 (3)	
C16	0.9326 (3)	0.33767 (10)	0.23824 (5)	0.0218 (3)	
C17	0.9758 (3)	0.37521 (11)	0.18767 (5)	0.0251 (3)	
H17	0.877 (4)	0.4283 (15)	0.1741 (7)	0.033 (5)*	
C18	1.1730 (3)	0.33647 (11)	0.15819 (6)	0.0271 (3)	
H18	1.201 (4)	0.3628 (13)	0.1225 (7)	0.027 (4)*	
C19	1.3197 (3)	0.26081 (11)	0.17784 (6)	0.0270 (3)	
H19	1.452 (4)	0.2330 (14)	0.1573 (7)	0.030 (5)*	
C20	1.2715 (3)	0.22127 (11)	0.22789 (6)	0.0248 (3)	
H20	1.374 (4)	0.1663 (14)	0.2398 (7)	0.029 (5)*	
C21	1.0792 (3)	0.26106 (10)	0.25795 (5)	0.0219 (3)	

### Atomic displacement parameters (Å^2^)

**Table:**

	*U*^11^	*U*^22^	*U*^33^	*U*^12^	*U*^13^	*U*^23^
O1	0.0187 (5)	0.0237 (5)	0.0284 (5)	0.0005 (4)	0.0011 (4)	−0.0042 (4)
O2	0.0264 (6)	0.0232 (5)	0.0365 (5)	−0.0060 (5)	0.0050 (5)	−0.0056 (4)
O3	0.0191 (5)	0.0341 (5)	0.0231 (4)	−0.0028 (5)	−0.0025 (4)	0.0014 (4)
O4	0.0169 (5)	0.0277 (5)	0.0210 (4)	−0.0022 (4)	−0.0003 (4)	0.0047 (4)
N1	0.0162 (5)	0.0243 (5)	0.0199 (5)	−0.0010 (5)	−0.0006 (4)	0.0006 (4)
C1	0.0166 (6)	0.0209 (6)	0.0219 (6)	−0.0012 (5)	0.0009 (5)	−0.0011 (5)
C2	0.0196 (6)	0.0214 (6)	0.0254 (6)	−0.0013 (5)	0.0031 (5)	0.0008 (5)
C3	0.0373 (10)	0.0340 (8)	0.0253 (7)	−0.0043 (7)	−0.0023 (6)	0.0050 (6)
C4	0.0577 (13)	0.0424 (10)	0.0287 (8)	−0.0021 (10)	−0.0003 (8)	0.0117 (7)
C5	0.0400 (10)	0.0229 (7)	0.0334 (7)	0.0015 (7)	0.0038 (7)	0.0012 (6)
C6	0.0197 (7)	0.0229 (6)	0.0203 (5)	−0.0006 (6)	−0.0005 (5)	−0.0009 (5)
C7	0.0177 (6)	0.0186 (6)	0.0222 (6)	−0.0001 (5)	0.0011 (5)	−0.0005 (4)
C8	0.0203 (7)	0.0250 (6)	0.0189 (5)	−0.0008 (6)	0.0003 (5)	0.0040 (5)
C9	0.0197 (6)	0.0244 (6)	0.0214 (6)	−0.0024 (5)	−0.0004 (5)	0.0024 (5)
C10	0.0221 (7)	0.0228 (6)	0.0233 (6)	−0.0024 (6)	−0.0008 (5)	−0.0009 (5)
C11	0.0298 (8)	0.0280 (7)	0.0239 (6)	−0.0033 (6)	0.0026 (6)	−0.0008 (5)
C12	0.0306 (8)	0.0310 (8)	0.0306 (7)	−0.0015 (7)	0.0065 (6)	−0.0053 (6)
C13	0.0287 (8)	0.0268 (7)	0.0367 (7)	0.0032 (7)	0.0011 (6)	−0.0055 (6)
C14	0.0292 (8)	0.0239 (7)	0.0287 (7)	0.0014 (6)	−0.0022 (6)	−0.0001 (5)
C15	0.0217 (7)	0.0215 (6)	0.0234 (6)	−0.0035 (6)	−0.0012 (5)	−0.0013 (5)
C16	0.0206 (7)	0.0215 (6)	0.0234 (6)	−0.0034 (5)	−0.0004 (5)	−0.0001 (5)
C17	0.0295 (8)	0.0228 (6)	0.0230 (6)	−0.0015 (6)	−0.0005 (6)	0.0018 (5)
C18	0.0322 (9)	0.0267 (7)	0.0224 (6)	−0.0064 (6)	0.0031 (6)	0.0012 (5)
C19	0.0245 (8)	0.0290 (7)	0.0274 (6)	−0.0037 (6)	0.0040 (6)	−0.0027 (5)
C20	0.0209 (7)	0.0257 (7)	0.0277 (6)	−0.0028 (6)	0.0008 (6)	0.0007 (5)
C21	0.0192 (7)	0.0233 (6)	0.0230 (6)	−0.0039 (6)	−0.0005 (5)	0.0012 (5)

### Geometric parameters (Å, °)

**Table:**

O1—C6	1.3270 (18)	C8—H8B	0.942 (17)
O1—H1H	0.88 (2)	C9—C10	1.518 (2)
O2—C6	1.2102 (18)	C9—C21	1.5196 (18)
O3—C7	1.2122 (17)	C9—H9	1.01 (2)
O4—C7	1.3471 (17)	C10—C11	1.385 (2)
O4—C8	1.4541 (16)	C10—C15	1.4092 (19)
N1—C7	1.3675 (17)	C11—C12	1.397 (2)
N1—C1	1.4484 (18)	C11—H11	1.005 (19)
N1—H1N	0.88 (2)	C12—C13	1.398 (2)
C1—C6	1.5284 (19)	C12—H12	0.98 (2)
C1—C2	1.5462 (19)	C13—C14	1.390 (2)
C1—H1	1.015 (19)	C13—H13	0.99 (2)
C2—C5	1.525 (2)	C14—C15	1.392 (2)
C2—C3	1.531 (2)	C14—H14	1.00 (2)
C2—H2	0.975 (19)	C15—C16	1.466 (2)
C3—C4	1.526 (2)	C16—C17	1.3957 (19)
C3—H3A	1.01 (2)	C16—C21	1.400 (2)
C3—H3B	1.01 (2)	C17—C18	1.393 (2)
C4—H4A	1.03 (3)	C17—H17	0.96 (2)
C4—H4B	1.00 (2)	C18—C19	1.390 (2)
C4—H4C	0.98 (3)	C18—H18	0.983 (18)
C5—H5A	1.02 (2)	C19—C20	1.399 (2)
C5—H5B	1.00 (2)	C19—H19	0.96 (2)
C5—H5C	1.00 (2)	C20—C21	1.387 (2)
C8—C9	1.529 (2)	C20—H20	0.98 (2)
C8—H8A	0.993 (19)		
			
C6—O1—H1H	108.7 (15)	C9—C8—H8B	111.2 (10)
C7—O4—C8	116.86 (11)	H8A—C8—H8B	108.6 (14)
C7—N1—C1	118.91 (12)	C10—C9—C21	102.23 (11)
C7—N1—H1N	115.3 (12)	C10—C9—C8	113.04 (12)
C1—N1—H1N	116.5 (12)	C21—C9—C8	108.49 (11)
N1—C1—C6	109.97 (11)	C10—C9—H9	113.8 (11)
N1—C1—C2	112.46 (11)	C21—C9—H9	111.1 (11)
C6—C1—C2	110.73 (11)	C8—C9—H9	108.0 (11)
N1—C1—H1	105.0 (11)	C11—C10—C15	120.39 (14)
C6—C1—H1	106.5 (10)	C11—C10—C9	129.63 (13)
C2—C1—H1	111.9 (11)	C15—C10—C9	109.96 (12)
C5—C2—C3	112.62 (14)	C10—C11—C12	118.80 (14)
C5—C2—C1	110.13 (12)	C10—C11—H11	119.3 (11)
C3—C2—C1	111.90 (12)	C12—C11—H11	121.9 (11)
C5—C2—H2	109.7 (11)	C11—C12—C13	120.76 (15)
C3—C2—H2	104.8 (10)	C11—C12—H12	118.6 (12)
C1—C2—H2	107.5 (11)	C13—C12—H12	120.6 (12)
C4—C3—C2	113.36 (15)	C14—C13—C12	120.59 (15)
C4—C3—H3A	111.4 (12)	C14—C13—H13	119.7 (12)
C2—C3—H3A	106.6 (13)	C12—C13—H13	119.7 (12)
C4—C3—H3B	109.2 (12)	C13—C14—C15	118.76 (14)
C2—C3—H3B	108.9 (12)	C13—C14—H14	121.6 (12)
H3A—C3—H3B	107.1 (19)	C15—C14—H14	119.6 (12)
C3—C4—H4A	110.2 (15)	C14—C15—C10	120.68 (13)
C3—C4—H4B	112.8 (13)	C14—C15—C16	130.69 (13)
H4A—C4—H4B	109 (2)	C10—C15—C16	108.64 (13)
C3—C4—H4C	110.1 (16)	C17—C16—C21	120.60 (13)
H4A—C4—H4C	111 (2)	C17—C16—C15	130.71 (14)
H4B—C4—H4C	104 (2)	C21—C16—C15	108.66 (12)
C2—C5—H5A	109.8 (12)	C18—C17—C16	118.24 (14)
C2—C5—H5B	109.5 (11)	C18—C17—H17	120.8 (12)
H5A—C5—H5B	106.7 (17)	C16—C17—H17	121.0 (12)
C2—C5—H5C	111.2 (13)	C19—C18—C17	121.20 (14)
H5A—C5—H5C	109.4 (17)	C19—C18—H18	121.0 (11)
H5B—C5—H5C	110.2 (17)	C17—C18—H18	117.7 (11)
O2—C6—O1	124.13 (13)	C18—C19—C20	120.52 (15)
O2—C6—C1	123.22 (13)	C18—C19—H19	121.3 (11)
O1—C6—C1	112.64 (12)	C20—C19—H19	118.1 (11)
O3—C7—O4	125.25 (12)	C21—C20—C19	118.54 (14)
O3—C7—N1	124.76 (13)	C21—C20—H20	123.1 (11)
O4—C7—N1	109.97 (12)	C19—C20—H20	118.4 (11)
O4—C8—C9	109.32 (11)	C20—C21—C16	120.86 (12)
O4—C8—H8A	107.3 (10)	C20—C21—C9	128.82 (13)
C9—C8—H8A	111.3 (10)	C16—C21—C9	110.31 (12)
O4—C8—H8B	109.0 (10)		
			
C7—N1—C1—C6	−88.14 (15)	C12—C13—C14—C15	0.7 (2)
C7—N1—C1—C2	147.96 (12)	C13—C14—C15—C10	0.6 (2)
N1—C1—C2—C5	−62.96 (16)	C13—C14—C15—C16	−179.33 (15)
C6—C1—C2—C5	173.56 (13)	C11—C10—C15—C14	−1.4 (2)
N1—C1—C2—C3	63.11 (16)	C9—C10—C15—C14	177.23 (13)
C6—C1—C2—C3	−60.37 (16)	C11—C10—C15—C16	178.54 (13)
C5—C2—C3—C4	−65.0 (2)	C9—C10—C15—C16	−2.87 (16)
C1—C2—C3—C4	170.27 (15)	C14—C15—C16—C17	1.9 (3)
N1—C1—C6—O2	−6.34 (19)	C10—C15—C16—C17	−178.03 (15)
C2—C1—C6—O2	118.56 (15)	C14—C15—C16—C21	179.97 (15)
N1—C1—C6—O1	174.17 (11)	C10—C15—C16—C21	0.08 (16)
C2—C1—C6—O1	−60.94 (15)	C21—C16—C17—C18	−1.9 (2)
C8—O4—C7—O3	0.8 (2)	C15—C16—C17—C18	176.00 (14)
C8—O4—C7—N1	179.33 (11)	C16—C17—C18—C19	1.5 (2)
C1—N1—C7—O3	−17.2 (2)	C17—C18—C19—C20	0.3 (2)
C1—N1—C7—O4	164.18 (11)	C18—C19—C20—C21	−1.8 (2)
C7—O4—C8—C9	121.17 (13)	C19—C20—C21—C16	1.4 (2)
O4—C8—C9—C10	−73.17 (14)	C19—C20—C21—C9	−179.33 (14)
O4—C8—C9—C21	174.19 (12)	C17—C16—C21—C20	0.5 (2)
C21—C9—C10—C11	−177.33 (15)	C15—C16—C21—C20	−177.88 (13)
C8—C9—C10—C11	66.3 (2)	C17—C16—C21—C9	−178.93 (13)
C21—C9—C10—C15	4.25 (15)	C15—C16—C21—C9	2.74 (16)
C8—C9—C10—C15	−112.17 (13)	C10—C9—C21—C20	176.47 (14)
C15—C10—C11—C12	0.9 (2)	C8—C9—C21—C20	−63.88 (19)
C9—C10—C11—C12	−177.41 (15)	C10—C9—C21—C16	−4.22 (15)
C10—C11—C12—C13	0.4 (2)	C8—C9—C21—C16	115.44 (13)
C11—C12—C13—C14	−1.2 (3)		

### Hydrogen-bond geometry (Å, °)

**Table:**

*D*—H···*A*	*D*—H	H···*A*	*D*···*A*	*D*—H···*A*
O1—H1H···O2^i^	0.88 (2)	1.77 (2)	2.6511 (14)	176 (2)
N1—H1N···O1^ii^	0.88 (2)	2.18 (2)	3.0433 (16)	167.6 (17)

Symmetry codes: (i) *x*−1/2, −*y*+1/2, −*z*+1; (ii) *x*+1, *y*, *z*.

## References

[bb1] Burla, M. C., Caliandro, R., Camalli, M., Carrozzini, B., Cascarano, G. L., De Caro, L., Giacovazzo, C., Polidori, G. & Spagna, R. (2005). *J. Appl. Cryst.* **38**, 381–388.

[bb2] Farrugia, L. J. (1997). *J. Appl. Cryst.* **30**, 565.

[bb3] Otwinowski, Z. & Minor, W. (1997). *Methods in Enzymology*, Vol. 276, *Macromolecular Crystallography*, Part A, edited by C. W. Carter Jr & R. M. Sweet, pp. 307–326. New York: Academic Press.

[bb4] Rigaku/MSC (2005). *CrystalClear* Rigaku/MSC Inc., The Woodlands, Texas, USA.

[bb5] Sheldrick, G. M. (2008). *Acta Cryst.* A**64**, 112–122. 10.1107/S0108767307043930 18156677

[bb6] Valle, G., Bonora, G. M. & Toniolo, C. (1984). *Can. J. Chem.* **62**, 2661–2666.

[bb7] Yamada, K., Hashizume, D. & Shimizu, T. (2008). *Acta Cryst.* E**64**, o1112. 10.1107/S1600536808014372 PMC2961627 21202623

[bb8] Yamada, K., Hashizume, D., Shimizu, T., Ohiki, S. & Yokoyama, S. (2008). *J. Mol. Struct.* In the press.

